# Enhancing Chemotherapy Efficacy: Investigating the Synergistic Impact of Paclitaxel and cd73 Gene Suppression on Breast Cancer Cell Proliferation and Migration

**DOI:** 10.7759/cureus.65027

**Published:** 2024-07-21

**Authors:** Farzaneh Hamidnia, Elif S Aslan, Souzan Najafi, Elham Baghbani, Sajjad Eslamkhah, Behzad Baradaran

**Affiliations:** 1 Molecular Biology and Genetics, Biruni University, Istanbul, TUR; 2 Medical Sciences, Immunology Research Center, Tabriz University, Tabriz, IRN

**Keywords:** mda-mb-231, cell migration, apoptosis, cell viability, breast cancer, cd73 sirna, paclitaxel, chemotherapy

## Abstract

Background

Enhancing chemotherapy efficacy is crucial in breast cancer treatment. This study examines the synergistic effects of paclitaxel, a common chemotherapeutic drug, and Cluster of differentiation 73 (*cd73)* gene suppression via siRNA on MDA-MB-231 breast cancer cells.

Methods

MDA-MB-231 cells were transfected with CD73 siRNA and treated with paclitaxel. Cell viability, apoptosis, and migration were assessed by using MTT assays, Annexin V-FITC/PI staining, and wound healing assays, respectively, with flow cytometry analyzing cell cycle distribution.

Results

The combination of CD73 siRNA and paclitaxel significantly reduced cell viability, lowering paclitaxel's IC50 from 14.73 μg/mL to 8.471 μg/mL, indicating enhanced drug sensitivity. Apoptosis rates increased with the combination treatment, while cell migration was significantly inhibited. Flow cytometry revealed cell cycle arrest in the Sub-G1 and G2-M phases.

Conclusion

These findings suggest that *cd73* gene suppression enhances paclitaxel's cytotoxic effects, promoting apoptosis and inhibiting cell migration in MDA-MB-231 breast cancer cell line. This combined strategy shows promise for improving breast cancer treatment outcomes by increasing the efficacy of existing chemotherapeutic regimens, warranting further research to explore its potential clinical applications and effectiveness in other breast cancer cell lines and models.

## Introduction

Breast cancer is a common malignancy among females, accounting for about one-third of cancers reported in women in the United States, and is the second leading cause of cancer-related deaths in women [1]. Although treatments like chemotherapy, surgery, radiation therapy, and immunotherapy are widely used, they have limitations such as recurrence, toxic side effects, high costs, and potential complications [2]. Therefore, new treatment modalities are urgently needed to overcome these challenges. MicroRNA (miRNA) therapy has emerged as a promising approach for treating breast cancer [3].

Recent years have seen the identification of small RNA regulators as essential components in cancer research. miRNAs are short, single-stranded RNA molecules, 19 to 24 nucleotides in length [2,4]. They do not encode genetic information, yet they are crucial in regulating gene expression after transcription in multicellular organisms. Research has revealed that miRNAs exhibit abnormal expression patterns in various tumors, including breast cancer [5]. The development of innovative treatment methods is imperative to address the challenges associated with breast cancer.

Small interfering RNA (siRNA), also known as short interfering RNA or silencing RNA, is a type of double-stranded RNA molecule, typically non-coding and about 20-24 base pairs long. Similar to miRNAs, siRNA functions in the RNA interference (RNAi) pathway by targeting and degrading mRNA post-transcription, preventing the translation of specific genes with complementary nucleotide sequences. This mechanism of gene silencing was first identified by Andrew Fire in 1998. This personalized approach holds great potential for optimizing treatment strategies and improving patient outcomes in breast cancer therapy [6, 7]. siRNA-*CD73* is a novel therapeutic approach that utilizes siRNA to target and inhibit the expression of *CD73* in converting extracellular adenosine monophosphate (AMP) to adenosine, which has immunosuppressive effects and promotes tumor growth. By specifically targeting *CD73* with siRNA, we can effectively suppress the expression and activity of this enzyme. This suppression leads to increased anti-tumor immune responses and reduced metastatic potential [7-10]. siRNA-*CD73* holds promise as a potential therapeutic strategy by disrupting the immunosuppressive tumor microenvironment and enhancing the effectiveness of existing anticancer therapies. Therapies that can also overcome the inhibitory effects of immune checkpoints are essential for improving patient outcomes in cancer treatment. Further research and clinical trials are underway to explore the full potential of siRNA-*CD73* as an innovative treatment option for cancer patients [8, 11, 12]. This study aimed to investigate the combined impact of paclitaxel, a widely used chemotherapy drug, and the suppression of the *cd73* gene on inhibiting the growth and migration of breast cancer cell lines.

## Materials and methods

Methods

This research took place at the Immunology Research Centre of Tabriz Medical University, Iran, in the context of the agreement between Biruni University (Istanbul, Türkiye) and Tabriz University of Medical Sciences Immunology Research Center (IRC). In this study, MDA-MB-231 (ATCC: HTB-26) breast cancer cell lines were obtained from the Iran National Cell Bank at the Pasteur Institute in Tehran, Iran. The cells were transfected with *CD73 *siRNA and treated with paclitaxel (14.73 µg/mL, Merck, USA). MTT assay was used (3-(4,5-Dimethylthiazol-2-yl)-2,5-Diphenyltetrazolium Bromide) to measure cell viability and checked for apoptosis with Annexin V-FITC/PI staining, and then assessed cell migration by using wound healing assays. Flow cytometry was also used to analyze the cell cycle.

Cell culture

The cells were cultured at 37°C for 48 h in Dulbecco's Modified Eagle Medium (DMEM) from Gibco, supplemented with 10% fetal bovine serum and antibiotics, specifically 100 μg/mL streptomycin and 100 IU/mL penicillin. The cultivation environment was maintained at 5% CO2 and 95% humidity to ensure optimal conditions. To promote healthy growth, the cells were routinely detached using 0.25% Trypsin-EDTA (Ethylenediaminetetraacetic Acid) and transferred to new culture dishes every 24-48 hours, once they reached 70-80% confluence in a monolayer formation [13, 14].

CD73 siRNA treatment

The cultured MDA-MB-231 cells were transfected with *CD73 *siRNA and a negative control siRNA (siRNA-control) (GenePharma Co, China). The transfection process utilized the Gene Pulser electroporation system (Bio-Rad, USA). An optimal dose of 100 pmol of the respective siRNAs was used for the transfection. Each transfection involved 1 × 10^6^ MDA-MB-231 cells suspended in 500 μL of electroporation buffer. The procedure was conducted at a voltage of 150 V for 12 milliseconds, following the manufacturer's protocol (Bio-Rad, USA). Post-transfection, the cells were cultivated in 6-well plates and observations were made every 24 and 48 hours to monitor their progress. At these intervals, cells were collected to determine the most effective timing and dosage for the siRNA treatment [7, 15].

Cell viability assay

To assess the impact of *CD73 *siRNA on the viability and chemosensitivity of MDA-MB-231 cells to carboplatin, the team utilized the MTT assay [16]. Initially, MDA-MB-231 cells transfected with *CD73 *siRNA were seeded into 96-well plates, using a density of 10,000 cells per well, and were allowed 24 hours (37°C) for cell adhesion and growth. Following this initial period, the cells were treated with various concentrations of carboplatin, from 10 µg/mL, 20 µg/mL, 50 µg/mL, 100 µg/mL, 200 µg/mL, 350 µg/mL, and incubated for an additional 24 hours. After this incubation period, the existing medium was replaced with an MTT solution (2 mg/mL), and the cells were incubated for another 4 hours to allow the MTT reaction to occur. To dissolve the formazan crystals that formed during the MTT assay, 200 μL of dimethyl sulfoxide (DMSO) was added to each well [17]. This was followed by a 30-minute incubation period to ensure complete solubilization of the crystals. Subsequently, the viability of the cells was quantified by measuring the absorbance at 570 nm using an ELISA reader from Tecan, Switzerland. To ensure consistency and reliability, this procedure was performed in triplicate.

Apoptosis assay

To evaluate apoptotic induction in the MDA-MB-231 breast cancer cell line, a double staining procedure was conducted using an Annexin V-FITC and propidium iodide (PI) kit (Exbio, Czech Republic). Initially, 3×10^5^ cells were cultured in six-well plates and subjected to various treatments: control, paclitaxel (IC25), paclitaxel (IC50), *CD73* siRNA, combined paclitaxel (IC25) with *CD73 *siRNA, and combined paclitaxel (IC50) with *CD73 *siRNA. After 24 hours of incubation, the cells were harvested and washed three times with phosphate-buffered saline (PBS) 1% (Merck, USA). The cells were then incubated in 500 µL of binding buffer containing 5 µL of FITC-conjugated Annexin V and 5 µL of PI at 37°C in darkness for 15 minutes. The stained cells were analyzed using the MACSQuant flow cytometer (Miltenyi Biotec, Germany). Data were processed with FlowJo software version 7.0.0 to quantify and assess apoptosis levels [18].

Colony formation test

After transfecting siRNA into the breast cancer cells, 6×10^3^ cells were cultured in 6-well plates. The cells were incubated at 37°C with 5% CO_2_ for 14 days, with the media being renewed every four days. After the incubation period, the cells were stained with crystal violet, and the colonies were imaged using an OPTICA inverted microscope (XDS-3, Italy). The colony formation was regularly monitored with the inverted microscope from the time of transfection up to 48 hours post-transfection. A graph was generated based on the data collected 48 hours after transfection [19].

Migration assay

To assess cell migration ability following transfection, a wound-healing assay was performed. Cells were cultured in a 24-well plate for 48 hours (37°C) and transfected with siRNAs when they reached 70-80% confluence. A wound was created in each well using a sterile yellow pipette tip, and cell debris was cleared using a serum-free medium. Images were taken at 0, 24-, and 48-hour post-transfection with an OPTICA inverted microscope system (XDS-3, Italy). All experiments were conducted three times to ensure the reliability and consistency of the results [11].

## Results

CD73 siRNA transfection sensitized MDA-MB-231 cells to paclitaxel treatment

**Figure 1:**
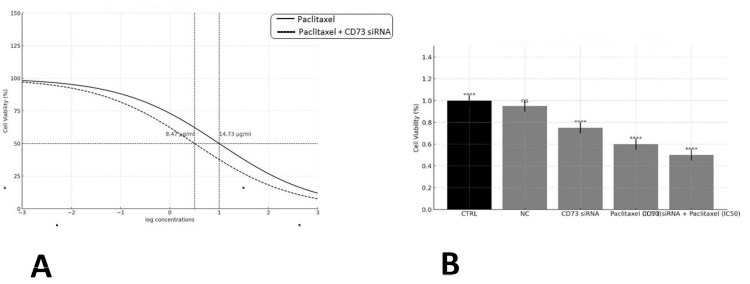
Effect of CD73 siRNA transfection on MDA-MB-231 cell viability (A) and sensitivity to paclitaxel treatment (B).

As mentioned, MTT testing was conducted to investigate the toxic impact of CD73 siRNA on breast cancer cells combined with carboplatin. The results indicated that CD73 siRNA transfection significantly (*p* < 0.001) decreased MDA-MB-231 cell viability compared to the controls. Meanwhile, no significant differences were observed in cell survival rates between the control and CD73 siRNA control groups (Figure 1A). Furthermore, as shown in Figure 1B, MTT results demonstrated that treating MDA-MB-231 cells with paclitaxel decreased cell survival in a dose-dependent manner, with the paclitaxel IC50 determined at 14.73 μg/mL. Pretreatment of MDA-MB-231 cells with CD73 siRNA significantly (*p* < 0.001) enhanced the cytotoxic effect of paclitaxel, lowering the IC50 value to 8.471 μg/mL.

Synergistic effect of CD73 siRNA and paclitaxel on apoptosis in MDA-MB-231 cells

**Figure 2:**
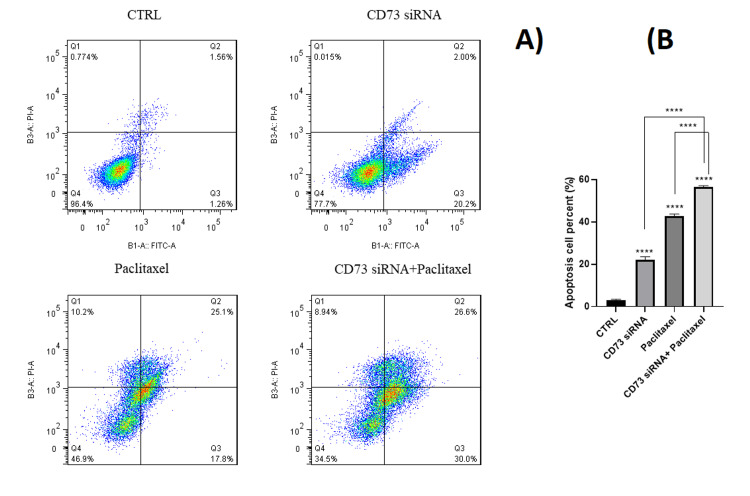
(A) Effects of CD73 siRNA knockdown and paclitaxel treatment on the apoptosis of MDA-MB-231 cells. The Annexin V-FITC/PI assay was used. The percentage of apoptotic cells has been provided. (CTRL: Control, CD73 siRNA: CD73 knockdown, paclitaxel: paclitaxel treatment, CD73 siRNA+paclitaxel: combined treatment). (B) The percentage of apoptotic cells has been provided. (*p < 0.05, **p < 0.01, ***p < 0.001, **p < 0.0001)

An Annexin V-FITC/PI double staining assay was conducted to evaluate whether CD73 siRNA induces apoptosis in MDA-MB-231 cells, either independently or in conjunction with paclitaxel. The findings revealed that the combined treatment elevated the rate of cell death to a similar extent as either CD73-targeting siRNA or paclitaxel alone. However, the level of apoptosis was significantly higher in the group treated with a combination of IC25/IC50 paclitaxel and siRNA compared to the cells treated with IC25/IC50 paclitaxel alone. This supports the MTT assay results (Figure 2).

CD73 siRNA and paclitaxel combination inhibited breast cancer cell migration

**Figure 3:**
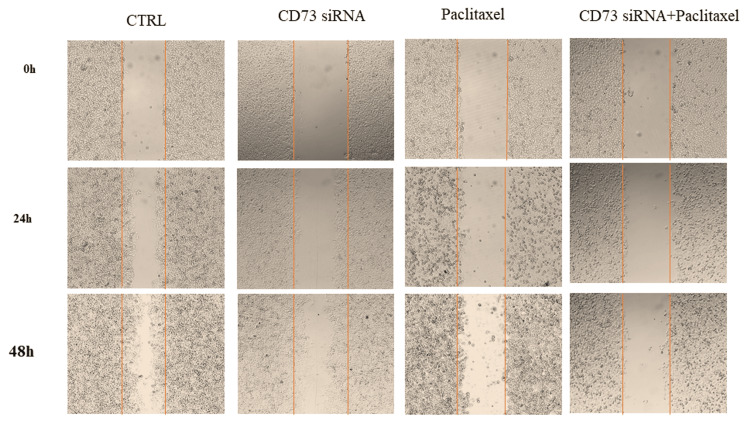
The impact of CD73 siRNA and paclitaxel on MDA-MB-231 cell migration. Representative images display the effects of CD73 siRNA, paclitaxel, and their combination at 0, 24, and 48 hours after treatment. (**p<0.01, ***p<0.001 compared to control)

Given that cancer cell migration and invasion are critical steps in metastasis, we investigated the impact of *cd73* knockdown via siRNA on the migration of MDA-MB-231 cells using a wound scratch assay. The results indicated that the down-regulation of *cd73* with specific siRNA or paclitaxel treatments alone significantly inhibited the migration and invasion of MDA-MB-231 cells. However, the combination of siRNA and paclitaxel markedly enhanced these inhibitory effects (Figure 3). These findings suggest that *cd73* plays a role in promoting tumor invasion and metastasis, and that combining CD73 siRNA with paclitaxel significantly diminishes the metastatic potential of these cells.

The effect of CD73 siRNA and their combined restoration on the clonogenicity and stemness of MDA-MB-231 cells

**Figure 4:**
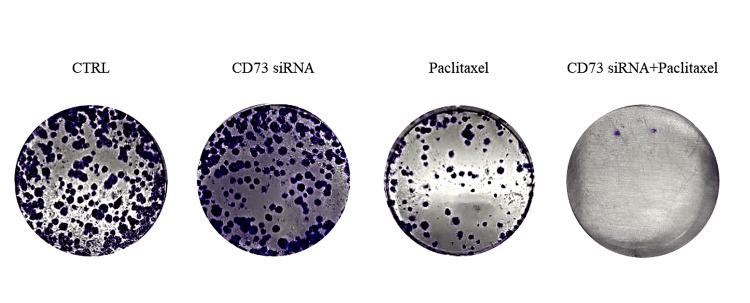
Effect of CD73 siRNA and paclitaxel on colony formation in MDA-MB-231 cells: comparison of monotherapies and combination treatment. This figure shows the colony formation of MDA-MB-231 cells following treatment with *CD73 *siRNA, paclitaxel, and their combination. The images correspond to different treatments and concentrations used: (A) Control, (B) *CD73 *siRNA, (C) Paclitaxel at 14.73 µg/mL, and (D) Combination of *CD73 *siRNA and paclitaxel at 8.47 µg/mL. The time of exposure was 48 hours for all treatments.

Our results demonstrated that both paclitaxel and CD73 siRNA significantly reduced colony numbers compared to the negative control. Additionally, the combination of CD73 siRNA and paclitaxel resulted in a more substantial decrease in colony numbers than either treatment alone (Figure 4). As a crucial factor in cancer stem cell behavior, *cd73* plays a pivotal role in these outcomes.

Combination of paclitaxel treatment and CD73 silencing induces Sub-G1 and G2-M cell cycle arrest

**Figure 5:**
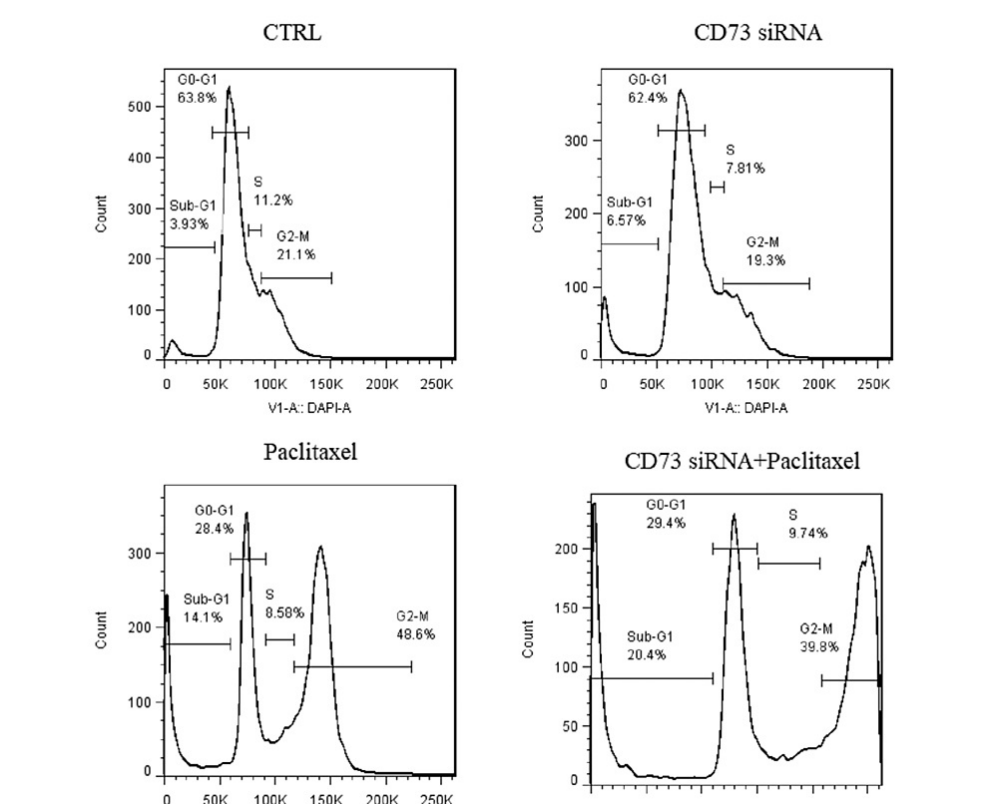
Distribution of MDA-MB-231 cells subjected to four different treatments: Control, CD73 siRNA, paclitaxel, and a combination of CD73 siRNA and paclitaxel. Both paclitaxel and CD73 siRNA individually induced cell cycle arrest. The combination treatment of CD73 siRNA and paclitaxel further intensified this effect, causing cell cycle arrest in the sub-G1 and G2-M phases.

After transfecting the siRNAs into the MDA-MB-231 breast cancer cells, we analyzed the cell cycle distribution for the control, CD73 siRNA, paclitaxel, and CD73 siRNA + paclitaxel treated groups. The control group exhibited a typical cell cycle distribution with 63.8% of cells in G0-G1, 3.93% in Sub-G1, 11.2% in S, and 21.1% in G2-M phase. CD73 siRNA treatment resulted in 62.4% of cells in G0-G1, 6.57% in Sub-G1, 7.81% in S, and 19.3% in G2-M, indicating a slight increase in the Sub-G1 phase. Paclitaxel treatment significantly altered the cell cycle, reducing the G0-G1 population to 28.4%, while increasing Sub-G1 to 14.1% and G2-M to 48.6%, suggesting mitotic arrest. The combination of CD73 siRNA and paclitaxel showed dramatic changes, with 29.4% of cells in G0-G1, 20.4% in Sub-G1, 9.74% in S, and 39.8% in G2-M, indicating a substantial increase in the Sub-G1 and G2-M phases. These results demonstrate that paclitaxel, both alone and in combination with CD73 siRNA, effectively induces cell cycle arrest, particularly in the G2-M phase (Figure 5).

## Discussion

Despite advancements in understanding breast cancer biology, there is a pressing need for new treatments that offer high efficacy and minimal side effects for affected individuals. Immune checkpoints can inhibit immune responses through a coinhibitory effect, making it crucial to find therapies that can overcome this challenge and improve patient outcomes [4, 20-22]. *cd73* is one such immune checkpoint that plays a significant role in the development and metastasis of breast cancer and this* *checkpoint significantly influences breast cancer development and metastasis through its enzymatic production of adenosine, which supports tumor growth and impairs immune surveillance [23]. *cd73* is commonly overexpressed in various cancers, including breast cancer. It contributes to an immunosuppressive tumor environment, aids tumor invasion, and promotes angiogenesis. Targeting *cd73* with siRNA is a promising therapeutic approach, as silencing its gene expression can disrupt these harmful processes [24]. These results indicate that CD73 siRNA is a key factor in improving the sensitivity of breast cancer cells to paclitaxel therapy.

Given the elevated expression of *cd73* in breast cancer, this study aimed to investigate the combined inhibitory effects of *cd73* gene suppression and paclitaxel on the MDA-MB-231 breast cancer cell line. The results of the MTT assay on MDA-MB-231 cells indicated that combining CD73 silencing with paclitaxel significantly reduced cell viability compared to paclitaxel alone. Additionally, the study demonstrated that *cd73* knockdown significantly enhances the sensitivity of MDA-MB-231 cells to paclitaxel chemotherapy. These findings align with previous studies showing that *cd73* suppression can increase the sensitivity of cancer cells to paclitaxel [25].

Annexin V/PI staining demonstrated that *cd73* silencing increases the apoptosis rate induced by paclitaxel in MDA-MB-231 cells. The results indicated that CD73-siRNA alone had a significant effect on cell viability and apoptosis rates compared to the control group. Remarkably, the apoptotic effect was synergistically enhanced when paclitaxel and *cd73* gene suppression were combined. These findings align with Liu et al.'s study, which showed that paclitaxel induces apoptosis in breast cancer cells [26]. Additionally, other researches demonstrated that *cd73* silencing reduces cell viability in murine cancer cells [25-27].

This study investigated the combined effect of cd73 siRNA and paclitaxel on the migration and cell cycle of MDA-MB-231 cells using flow cytometry. Results showed that both treatments led to significant cell cycle arrest, with paclitaxel predominantly causing arrest in the G2-M phase and the combination treatment leading to more cells in the sub-G1 phase, indicating increased apoptosis. Paclitaxel stabilizes microtubules, disrupting mitotic processes and causing cell death, while cd73, an enzyme in the adenosine signaling pathway, promotes tumor growth by inhibiting immune responses and supporting angiogenesis. Silencing cd73 with siRNA disrupts these pathways, leading to increased apoptosis. The combination treatment's enhanced apoptotic effect suggests a synergistic interaction, making cells more susceptible to paclitaxel. These findings align with previous studies showing cd73 knockdown reduces cancer cell migration and clonogenicity [12, 28], supporting Gao et al.'s findings on cd73's role in tumor growth and immune escape. Thus, combining paclitaxel with cd73 inhibition presents a promising therapeutic strategy, warranting further research to understand the underlying molecular mechanisms and confirm its benefits [29].

This approach not only shows potential for inhibiting tumor growth but also for enhancing the immune system's ability to fight cancer, making cd73 siRNA a valuable addition to breast cancer treatment strategies. However, the current research has some limitations.

In this research, we only investigated the impact of combining paclitaxel with *cd73* silencing, without exploring the effects of other chemotherapy drugs such as doxorubicin or cisplatin. It also primarily focused on migration and cell cycle processes, neglecting other potential mechanisms or pathways. These limitations indicate the need for further research to validate and expand the findings.

## Conclusions

Our study investigated the combined effect of *cd73 *gene suppression and paclitaxel chemotherapy on MDA-MB-231 breast cancer cells. The results demonstrated that combining *cd73 *silencing with paclitaxel significantly reduced cell viability, arrested cell growth, and decreased cell migration. These findings align with recent research, which highlights the importance of targeting the cd73 gene, as its suppression can significantly reduce tumor progression and increase cell death in breast cancer cells. This combination therapy appears to be a promising new approach for treating breast cancer. However, concerns about the effectiveness of immune checkpoint inhibitors in cancer therapy persist, emphasizing the need for further research to validate and expand these results.
